# The *mbo* Operon Is Specific and Essential for Biosynthesis of Mangotoxin in *Pseudomonas syringae*

**DOI:** 10.1371/journal.pone.0036709

**Published:** 2012-05-17

**Authors:** Víctor J. Carrión, Eva Arrebola, Francisco M. Cazorla, Jesús Murillo, Antonio de Vicente

**Affiliations:** 1 Instituto de Hortofruticultura Subtropical y Mediterránea “La Mayora” (IHSM-UMA-CSIC), Departamento de Microbiología, Facultad de Ciencias, Universidad de Málaga, Málaga, Spain; 2 Instituto de Hortofruticultura Subtropical y Mediterránea “La Mayora” (IHSM-UMA-CSIC), Estación Experimental La Mayora, Málaga, Spain; 3 Laboratorio de Patología Vegetal, ETS de Ingenieros Agrónomos, Universidad Pública de Navarra, Pamplona, Spain; University of the West of England, United Kingdom

## Abstract

Mangotoxin is an antimetabolite toxin produced by certain *Pseudomonas syringae* pv. syringae strains. This toxin is an oligopeptide that inhibits ornithine N-acetyl transferase, a key enzyme in the biosynthesis of ornithine and arginine. Previous studies have reported the involvement of the putative nonribosomal peptide synthetase MgoA in virulence and mangotoxin production. In this study, we analyse a new chromosomal region of *P. syringae* pv. syringae UMAF0158, which contains six coding sequences arranged as an operon (*mbo* operon). The *mbo* operon was detected in only mangotoxin-producing strains, and it was shown to be essential for the biosynthesis of this toxin. Mutants in each of the six ORFs of the *mbo* operon were partially or completely impaired in the production of the toxin. In addition, *Pseudomonas* spp. mangotoxin non-producer strains transformed with the *mbo* operon gained the ability to produce mangotoxin, indicating that this operon contains all the genetic information necessary for mangotoxin biosynthesis. The generation of a single transcript for the *mbo* operon was confirmed and supported by the allocation of a unique promoter and Rho-independent terminator. The phylogenetic analysis of the *P. syringae* strains harbouring the *mbo* operon revealed that these strains clustered together.

## Introduction

*Pseudomonas syringae* is a plant-pathogenic bacterium that infects a wide variety of plants and produces several phytotoxic compounds [1], [2], [3], [4]. The phytotoxins produced by *P. syringae* pathovars are important for virulence and symptom production [5]. Although not essential for pathogenicity, these toxins generally act as virulence factors of the *P. syringae* strains and are involved in the disease symptom development in many plant diseases [1], [4], [6]. The phytopathogenic *P. syringae* pv. syringae strains can produce two types of necrosis-inducing lipopeptide phytotoxins, syringomycins and syringopeptins. Both phytotoxins are amphipathic molecules composed of a hydrophobic 3-hydroxy carboxylic acid tail of varying lengths and a charged cyclic peptide head [7], [8], [9]. It is the amphipathic nature of these phytotoxins that enables them to insert into membranes and form pores that ultimately lead to plant cell death and necrosis [9], [10]. Both types of toxins are synthesised separately by modular nonribosomal peptide synthetases [11], [12], [13]. The non-ribosomal peptide synthases (NRPSs) catalyse the activation and addition of amino acids into the peptide chain [3], [14], [15], [16]. The genes dedicated to the biosynthesis, secretion, and the genes responsible for the regulation of these toxins are located in the syringomycin and syringopeptin gene clusters, which are adjacent to one another on the chromosome [13], [17]. Another phytotoxin group is the chlorosis-inducing non-host-specific phytotoxin coronatine. This phytotoxin is produced by several pathovars of *P. syringae*, including pvs. atropurpurea, glycinea, maculicola, morsprunorum, and tomato [1], [18], [19]. Coronatine also acts as a virulence factor [20], that promotes the bacterial entry into the plant host by stimulating the opening of the stomata [21] and suppressing salicylic acid-dependent host defences [22], [23]. The genetic basis for coronatine production was first identified in *P. syringae* pv. glycinea PG4180 as a 32.8 kb *cor* cluster on the plasmid p4180 [24]. In other strains, such as *P. syringae* pv. tomato DC3000, the *cor* genes are derived from only the chromosome where they are co-localised with other virulence genes, including two clusters of effector genes [25]. However, in many strains of *P. syringae*, the *cor* cluster is usually located on large (80–110 kb) indigenous plasmids belonging to the pPT23A family, and could be transferred via conjugation between strains [26], [27].

The last group of phytotoxins are those classified as antimetabolite toxins. They are generally small-sized metabolites that exhibit strong inhibitor effects in plant cells by causing an increase in disease symptoms; therefore, they are considered as virulence factors [1], [6]. Currently, each antimetabolite toxin described has specific target enzymes involved in the glutamine and arginine biosynthesis pathways of the host, enhancing disease symptoms and increasing the virulence of the bacterial toxin-producing pathogen [6], [28]. The best established antimetabolite toxins are tabtoxin and phaseolotoxin [1], [3], [4]. Tabtoxin consists in tabtoxine-β-lactam and threonine. Tabtoxin is associated with the symptoms of wildfire disease in tobacco. This toxin is produced by strains of *P. syringae* pv. tabaci, pv. coronafaciens and pv. garcae [29] that irreversibly inhibit glutamine synthetase. Diverse studies have demonstrated that tabtoxin biosynthesis proceeds along the lysine pathway (*dabABCDE*), branching off after tetrahydropicolineate and before diaminopimelate formation [4], [30], [31]. The biosynthetic enzymes are encoded by the 15 kb *tab/tbl* gene cluster [32]. The GacS/GacA two-component system has been reported to be an important regulatory genes in *P. syringae* pv. coronafaciens, due to its crucial role in the transcription of the *tblA* gene [1], [33]. On the other hand, phaseolotoxin is a sulphodiaminophosphinyl moiety linked to a tripeptide consisting of ornithine, alanine and homoarginine [1], [3], [4]. This toxin is produced primarily by strains of *P. syringae* pv. phaseolicola, pv. syringae and pv. actinidae and causes chlorosis by inhibiting the enzymatic activity of ornithine carbamoyltransferase in host plants [1], [34], [35]. It has been demonstrated that the homoarginine and ornithine residues of phaseolotoxin are synthesised by a transamidination reaction from arginine and lysine [28], [36]. The 28 kb phaseolotoxin gene cluster, termed the *argK–tox* cluster, is composed of 23 genes arranged into five main transcriptional units, including two monocistronic (*argK* and *phtL*) and three polycistronic operons (*phtA*, *phtD* and *phtM*) [35]. A nonribosomal thiotemplate mechanism might be required for the synthesis of phaseolotoxin similar to the mechanism described for the biosynthesis of the other antimetabolite toxins [3], [37], [38].

Our group reported *P. syringae* pv. syringae as the causal agent for the bacterial apical necrosis of the mango [39]. More than 87% of the *P. syringae* pv. syringae strains isolated from mango tissues were demonstrated to produce mangotoxin [40], which is an antimetabolite toxin that inhibits ornithine N-acetyl-transferase, a key enzyme in the biosynthetic pathway of ornithine and arginine [37], [40]. The production of mangotoxin has been analysed using *P. syringae* pv. syringae UMAF0158 as a bacterial model. At the moment, the production of mangotoxin has been strongly associated with strains belonging to pv. syringae [40]. However, recent studies have reported that mangotoxin is produced in the phaseolotoxin-producing strain *P. syringae* pv. syringae CFBP 3388 and by *P. syringae* pv. avellanae strains [41]. The preliminary characterisation of mangotoxin suggested that it was a small oligopeptide (two or three amino acids), similar to the well-known antimetabolite toxins, tabtoxin or phaseolotoxin [1], [3], [4]. Mangotoxin is secreted in the media as a hydrophilic molecule of approximately 3 kDa in size that is sensitive to proteases but resistant to extreme pH and high temperatures [40]. Mangotoxin acts as a virulence factor that increases the disease symptoms of *P. syringae* pv. syringae strains during infection [6], [37]. The genetic basis of the production of mangotoxin has been recently studied. A putative NRPS gene (*mgoA*), was suggested to be necessary for the production of mangotoxin and in the virulence of *P. syringae* pv. syringae [37]. *mgoA* is a gene in the *mgo* operon (mangotoxin generating operon). This operon is 5,779 bp in size and comprises four genes, *mgoB, mgoC, mgoA* and *mgoD*, flanked by a promoter and terminator region [42]. Recent studies focusing on the *P. entomophila pvf* gene cluster, which is homologous to the *mgo* operon, suggest that this gene cluster serves as a regulator of virulence factors in pathogenic strains of *Pseudomonas* spp. [43].

In this work, we describe a biosynthetic group of specific and essential genes involved in the production of mangotoxin in *P. syringae* strains. We show that these genes are arranged into an operon that we call *mbo* and which is composed of six genes. The genetic analysis of the *mbo* operon revealed the presence of a promoter and a Rho independent terminator that produce a unique polycistronic transcript. The role of each gene in the production of mangotoxin was determined using insertional mutagenesis and complementation experiments. Furthermore, we report that the *Pseudomonas* mangotoxin non-producing strains could be converted to producers upon transformation with the *mbo* operon. Finally, we demonstrate that *Pseudomonas* strains that contain *mbo* genes are strongly clustered together, suggesting that the *mbo* operon was acquired one time during the evolution and was present in a common ancestor.

## Results

### Different Genes are Involved in the Production of Mangotoxin

**Figure 1:**
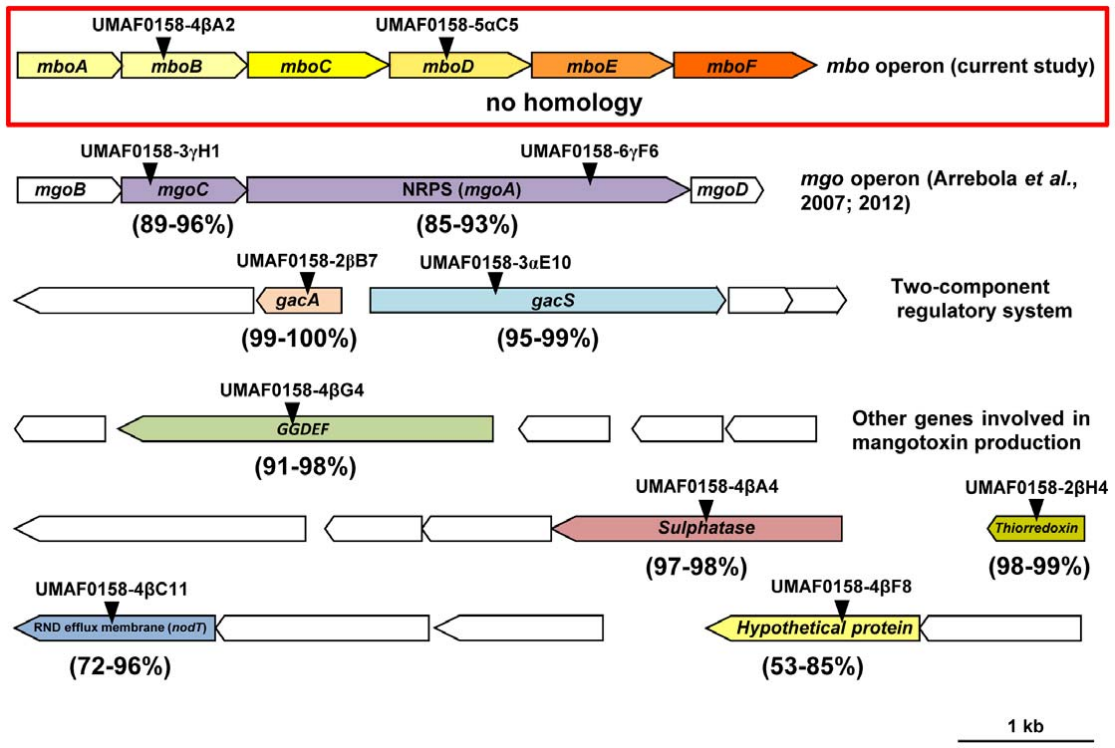
Allocation of the mini*Tn5* insertion in the eleven selected derivative mutants affected in mangotoxin production in the *P. syringae* pv. syringae UMAF0158 wild-type strain. The insertion point of each mutant (▾) was characterised by analysis of the flanking regions of the mini*Tn5* insertion. The identity analysis was performed by comparing the sequenced genomes of *P. syringae* pv. syringae B728a, pv. phaseolicola 1448A and the pv. tomato DC3000 (the amino acid identity range is shown into brackets under each gene). The *mbo* operon involved in mangotoxin biosynthesis is indicated with a red box, and no identity was found. The *mgo* operon, the two-component regulatory system (*gacA* and *gacS*) and other genes that are involved in the production of mangotoxin are also shown.

A previously constructed mini*Tn5* random mutant collection [37] was used to identify genes involved in mangotoxin production. We uncovered eleven mutants that were arrested in the production of mangotoxin. The disrupted sequences of each mini*Tn5* mutant were analysed, showing insertions in different *P. syringae* pv. syringae UMAF0158 coding sequences (Figure 1). Two mini*Tn5* mutants (UMAF0158-5αC5 and UMAF0158-4βA2) were selected due to the absence of sequence identity with other published *Pseudomonas spp.* sequences. Initially, these genes were assumed to be specific to UMAF0158 (red box, Figure 1 and S1). An additional two mini*Tn5* mutants (UMAF0158-3γH1 and UMAF0158-6γF6) were located in the *mgo* operon. One of these mutants had a disruption in a putative NRPS (*mgoA*); the involvement of this gene in the production of mangotoxin has been previously demonstrated [37], [42]. The analysis of the other remaining mutants showed the involvement of genes encoding the two-component regulatory system (GacS/GacA) and other orthologous genes present in the published genomes of *Pseudomonas* spp., such as the GGDEF domain protein (UMAF0158-4βG4), thioredoxin (UMAF0158-2βH4), sulphatase (UMAF0158-4βA4), the RND efflux membrane protein (UMAF0158-4βC11) and a hypothetical protein (UMAF0158-4βF8, Figure 1).

**Figure 2:**
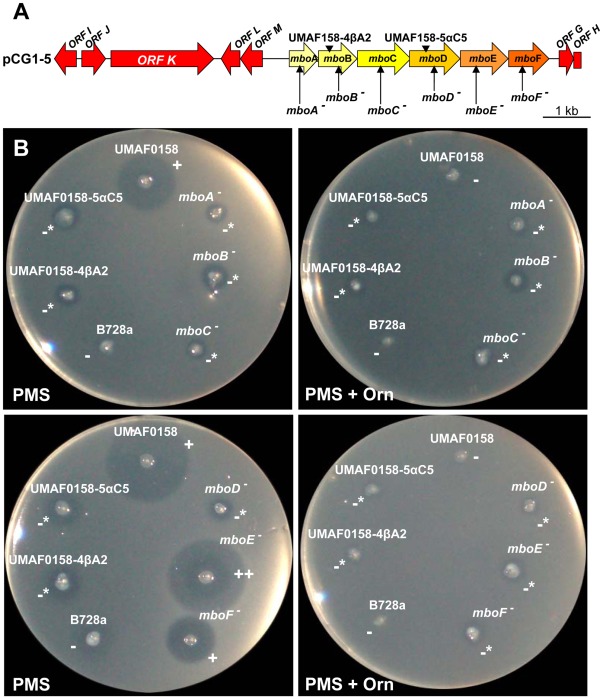
Characterisation of the mutants in the *mbo* operon. (**A**) Chromosome region cloned into pCG1-5 containing the mbo operon (*mboA* to *F*) and the location of insertional and mini*Tn5* mutants used in this study. The vector pCG1-5 contains a 12,510 bp insert of chromosomal DNA derived from the wild-type strain *P. syringae* pv. syringae UMAF0158 (JQ409468). The derivative strains constructed using site-directed mutagenesis (↑) or mini*Tn5* (▾) insertion are indicated. The flanking genes of the *mbo* operon are indicated in red. (**B**) The bioassay for the production of mangotoxin using derivative insertional mutants in the different genes of the *mbo* operon was evaluated by stabbing the strains on minimal medium PMS supplemented or not with ornithine. The *P. syringae* pv. syringae UMAF0158 and B728a strains were used as positive and negative controls respectively. The results are indicated as follows: - absence of inhibition halo, + presence of inhibition halo, -* slight toxic production which did not reverts with ornithine. The toxic activity, which reverts in the presence of ornithine, denotes the production of mangotoxin.

In this study, we focus on the analysis of the coding sequences that are disrupted in the mutants UMAF0158-5αC5 and UMAF0158-4βA2 (Figure 1) due to non-homology with other orthologous genes present in the completely sequenced and annotated genome of the three *P. syringae* model strains (pv. syringae B728a, pv. phaseolicola 1448A and pv. tomato DC3000). A genomic library of *P. syringae* pv. syringae UMAF0158 was screened for the disrupted sequences of the mutants UMAF0158-5αC5 and UMAF0158-4βA2, and these sequences were located in the pCG1-5 genomic clone (JQ409468). The analysis of the genomic clone pCG1-5 revealed the presence of 13 ORFs (Figure 2A); six of these ORFs were specific for the strain UMAF0158 (Figure S1). These six ORF-specific genes were named *mboA*, *B*, *C*, *D*, *E* and *F* in accordance with the mangotoxin biosynthetic operon. The sequences that were disrupted in the mangotoxin-defective mutants UMAF0158-5αC5 and UMAF0158-4βA2 were located in these six ORF-specific genes (Figure 1 and 2A) and were absent in other sequenced *Pseudomonas* species. In addition, the genomic clone pCG1-5 was able to restore the production of mangotoxin in the mini*Tn5* mutants UMAF0158-5αC5 and UMAF0158-4βA2.

### The Role of Specific *mbo* Genes in the Production of Mangotoxin

The sequencing of the pCG1-5 genomic clone showed that seven (ORFs G to M) of the thirteen ORFs were present in the genome of other *Pseudomonas* spp., such as *P. syringae* pv. syringae B728a (Figure S1). The six remaining ORFs were considered specific for the production of mangotoxin (*mboA* to *mboF*). To determine if these six genes were involved in mangotoxin production, insertional mutants were obtained for each gene (Figure 2), and mangotoxin production was analysed. The disruption of the *mboA*, *mboB*, *mboC* and *mboD* genes resulted in derivative mutants (*mboA* ***^−^***, *mboB* ***^−^***, *mboC* ***^−^*** and *mboD* ***^−^***) that were completely unable to produce mangotoxin (Figure 2B and Table 1). Mutation of the *mboE* and *mboF* genes (*mboE* ***^−^*** and *mboF* ***^−^***) resulted in altered mangotoxin production compared with that of the wild-type strain (Figure 2B and Table 1). The relative levels of mangotoxin production in the random and insertional mutants were compared with that of the wild-type strain *P. syringae* pv. syringae UMAF0158 (Table 1). The wild-type strain maintained mangotoxin production until a dilution of 1∶8. However, the mini*Tn*5 random and insertional mutants *mboA* ***^−^***, *mboB* ***^−^***, *mboC* ***^−^*** and *mboD* ***^−^*** did not show mangotoxin production. The insertional mutants *mboE* ***^−^*** and *mboF* ***^−^*** retained a slight ability to produce mangotoxin (detectable up to a dilution of 1∶2). The insertional mutants were verified using PCR, sequencing and Southern blot analysis. Further RT-PCR experiments with the six insertional mutants from *mboA* to *mboF* genes showed the presence of transcripts up and downstream of the interrupted gene (Figure S2). To study the restoration of mangotoxin production, pLac-AF and pLac-FA were constructed (Figure S3). These plasmids comprised the genomic sequences from *mboA* to *mboF* cloned into the pBBR1MCS-5 vector. The complemented mutants harbouring the plasmid pLac-AF, with a complete sequence corresponding to the interrupted sequence, resulted in the complete restoration of mangotoxin production, with an even slightly higher production of mangotoxin than the wild-type strain (Table 1).

**Table 1:** *E. coli* growth inhibition test.

Bacterial strains	Mangotoxin production	Dilutions of cultures filtrates^a^					
		1∶1	1∶2	1∶4	1∶8	1∶16	+ Orn
*Wild type*							
UMAF0158	+	21.9±0.4	18.2±0.4	13.9±0.4	9.5±0.5	<9	<9
*miniTn5 mutants*							
UMAF0158-4βA2	−	<9	<9	<9	<9	<9	<9
UMAF0158-5αC5	−	<9	<9	<9	<9	<9	<9
*Insertion mutants*							
*mboA* ***^−^***	−	<9	<9	<9	<9	<9	<9
*mboB* ***^−^***	−	<9	<9	<9	<9	<9	<9
*mboC* ***^−^***	−	<9	<9	<9	<9	<9	<9
*mboD* ***^−^***	−	<9	<9	<9	<9	<9	<9
*mboE* ***^−^***	+	21.3±0.5	15.3±0.5	<9	<9	<9	<9
*mboF* ***^−^***	+	23.0±1.0	15.0±1.0	<9	<9	<9	<9
*pLac-AF transformation in*							
UMAF0158-4βA2	*+*	23.0±1.0	17.0±1.0	13.3±1.0	10.5±0.5	<9	<9
UMAF0158-5αC5	*+*	22.0±1.0	16.3±1.5	12.6±1.5	9.8±0.3	<9	<9
*mboA* ***^−^***	+	24.8±2.2	21.6±2.9	18.5±3.4	14.6±3.3	11.0±1.6	<9
*mboB* ***^−^***	+	27.3±0.5	24.3±0.5	21.6±1.1	17.6±0.5	13.3±1.5	<9
*mboC* ***^−^***	+	26.0±1.4	22.5±1.8	18.5±1.2	15.0±1.4	12.8±1.4	<9
*mboD* ***^−^***	+	26.0±0.6	20.3±1.0	20.3±0.5	15.8±0.7	13.1±0.7	<9
*mboE* ***^−^***	+	25.8±1.1	22.0±1.2	19.1±0.5	15.8±1.8	12.0±0.6	<9
*mboF* ***^−^***	+	27.0±1.0	22.0±1.0	18.0±1.7	15.3±0.5	11.3±0.5	<9

Specific inhibition by cell-free culture filtrates of *P. syringae* pv. syringae UMAF0158 and the derivative mini*Tn5* and insertion mutants strains grown in liquid minimal medium (PMS).

a Toxic activity is expressed as the diameter of the inhibition zone (in mm). The average and standard deviation values were obtained from three replicates of three experiments.

### The *mbo* Operon is Essential for Mangotoxin Production in *P. syringae*

The sequence containing the *mbo* genes, including the putative regulatory sequences (promoter and terminator), was cloned into the pBBR1MCS-5 vector [44]. Two plasmids were originated in the two orientations and were designated as pLac-AF and pLac-FA (Figure S3). The two constructs were individually able to complement mangotoxin production in the defective mutants. These two plasmids were transformed into other genetic backgrounds of non-producing mangotoxin strains that belong to different species of *Pseudomonas* spp. (Table 2). The transformation with pLac-AF allowed mangotoxin production in all transformed strains. However, the pLac-FA transformation allowed the production of mangotoxin in only strains belonging to the pathovars of *P. syringae*. The transformation of pLac-FA in *P. fluorescens* Pf0-1 [45] and Pf-5 [46] did not result in the production of mangotoxin (Table 2).

**Table 2:** Production of mangotoxin in different genetic backgrounds of *Pseudomonas spp.* transformed with the vectors pLac-AF (constitutive expression of *mbo* genes) and pLac-FA (own expression of *mbo* genes).

Strains	Transformed with:		
	None	pLac-AF	pLac-FA
*P. fluorescens*			
Pf-5	−	+	−
Pf0-1	−	+	−
*P. syringae* pv. syringae			
B728a	−	+	+
FF5	−	+	+
UMAF0158	+	+	+
*P. syringae* pv. phaseolicola			
1448A	−	+	+
*P. syringae* pv. tomato			
DC3000	−	+	+
PT23	−	+	+

### The *mbo* Operon is Transcribed as a Polycistronic mRNA

**Figure 3:**
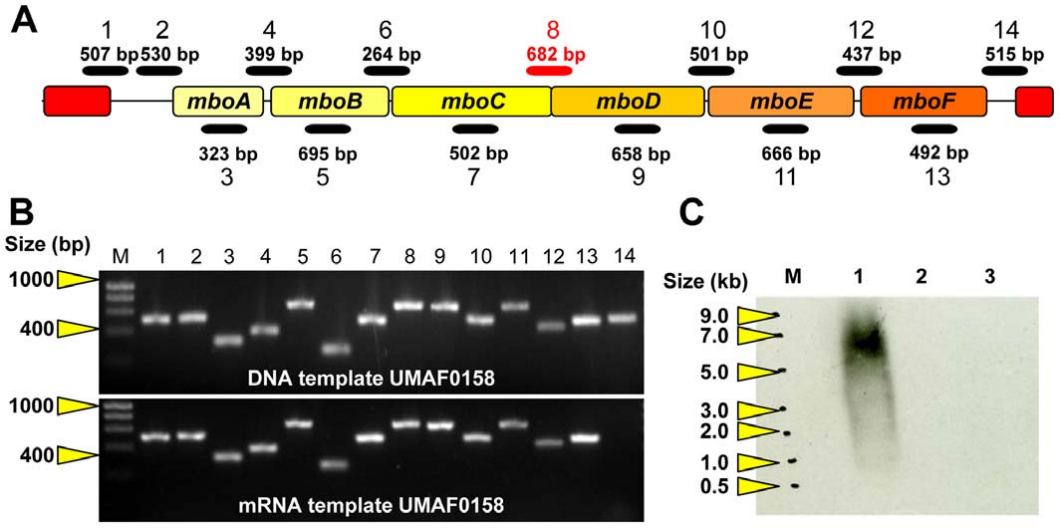
*mbo* operon transcriptional characterisation. (**A**) Schematic representation of the location and size of the amplified region obtained during the RT-PCR experiments. (**B**) PCR products from RT-PCR experiments using genomic DNA and mRNA as templates obtained from wild-type UMAF0158 and processed after 48 h of incubation at 22°C on liquid minimal medium PMS. The primer pairs used is detailed in Table S2. (**C**) Northern blot analysis of total RNA obtained from wild-type UMAF0158 and mutants in *gacA* and *mgoA* genes, using a DNA fragment between *mboC* and *mboD* genes as probes (shown in red in panel A). Lane M, ssRNA ladder; lane 1, UMAF0158; lane 2, *gacA* ***^−^*** (UMAF0158-2βB7) and lane 3, *mgoA* ***^−^***.

RT-PCR analyses revealed the co-transcription of the genes *mboA, mboB, mboC, mboD*, *mboE* and *mboF* and were confirmed by the amplification of the connecting areas located between the sequential ORFs and the internal regions of the transcript from the putative *mbo* operon (Figure 3A and B). The amplifications of the genomic DNA, which was used as a control, and the *mbo* mRNA from wild-type UMAF0158 were identical, except for the last amplification (Figure 3B lane 14), which suggests the presence of a putative transcript terminator. Thus, these results indicated the co-transcription of the six genes, forming a single polycistronic mRNA molecule.

The hybridisation analysis of the *mbo* operon transcript using total mRNA from wild-type UMAF0158 and the mini*Tn5* mutants UMAF0158-2βB7 and UMAF0158-6γF6 (*gacA* and *mgoA* mutants, respectively) showed the presence of a 6 to 7 Kb transcript in the line correspondent with the wild-type, which is consistent with the expected results (Figure 3C). In agreement with our previous results [37], this transcript was not detected in the non-mangotoxin-producing *gacA* ***^−^*** and *mgoA* ***^−^*** mutants.

### A Strong Promoter is Allocated Upstream *mbo* Genes

**Figure 4:**
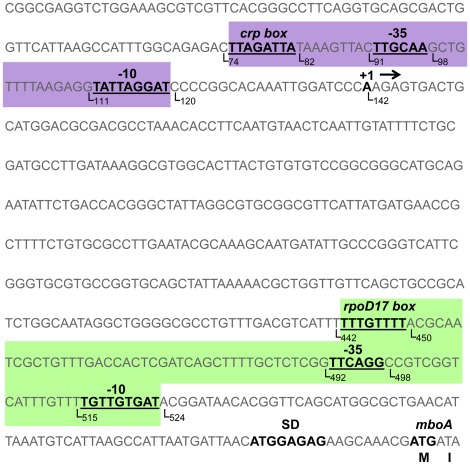
*mbo* operon promoter region. The non-coding region (603 bp), which contains the promoter sequences of the *mbo* operon. The nucleotide sequences of putative promoter P*_mboI_* and P*_mboII_* (indicated in purple and green, respectively) showing the proposed -10 box, -35 box and the TF binding sites; the nucleotide position is also indicated. The putative SD sequence of *mboA* is shown in bold type. The position of the first nucleotide to be transcribed was determined by 5′-RACE experiments and labelled as +1 in bold type; the transcription direction is also indicated (→).

**Figure 5:**
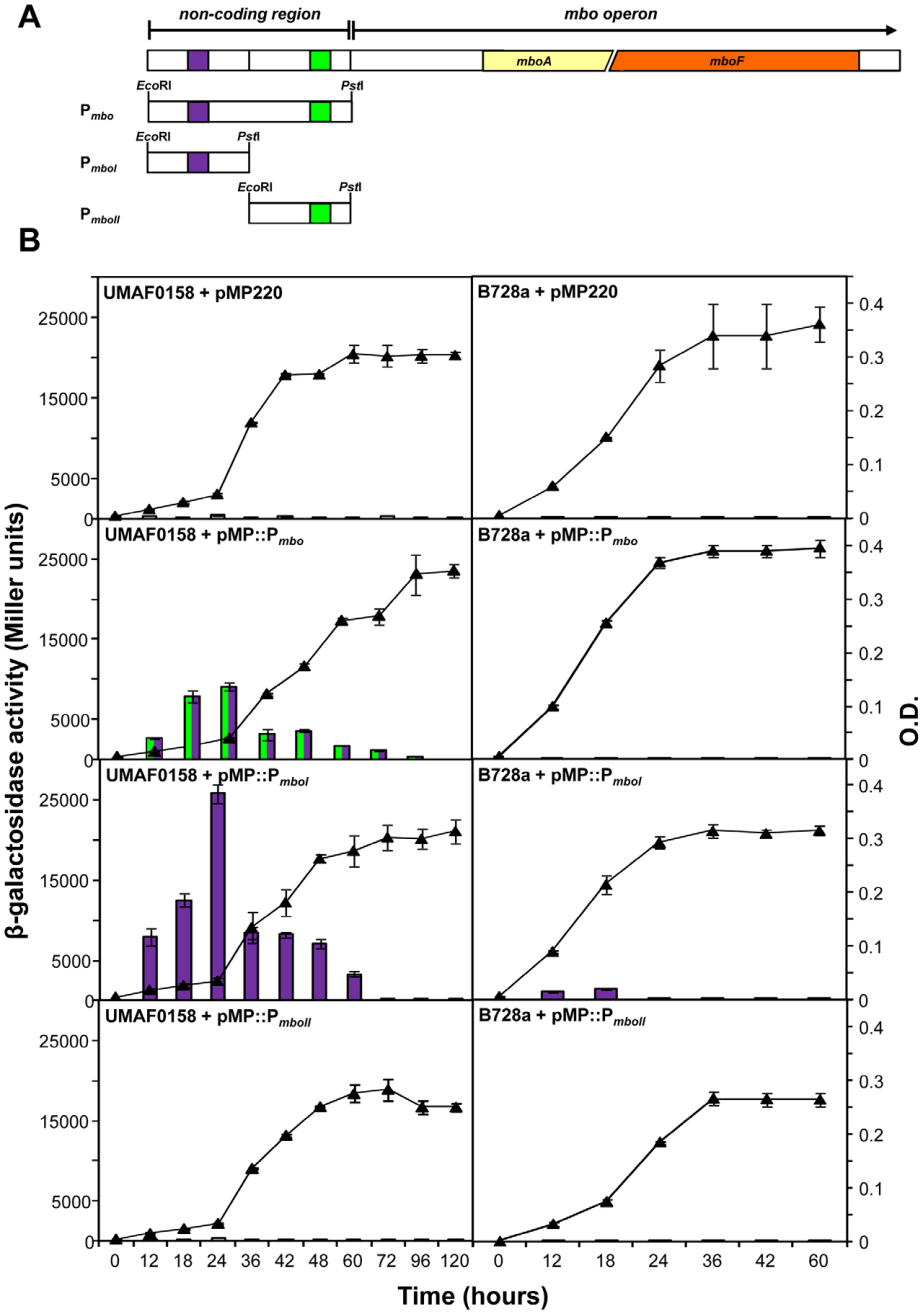
Determination of the promoter activity using the β-galatosidase assay. (**A**) Diagram of the DNA inserts cloned into pMP220 vector in every case assayed. P*_mbo_* construction included both putative promoters detected by bioinformatic analysis. P*_mboI_* construction only contains the activity corresponding to the first putative promoter (purple), and P*_mboII_* include the second putative promoter alone (green). (**B**) β- galatosidase expressed activity (bars diagrams) and optical density (line) of the culture on minimal medium PMS of wild-type strains of *P. syringae* pv. syringae UMAF0158 and *P. syringae* pv. syringae B728a. These strains were transformed with pMP::P*_mbo_*, pMP::P*_mboI_,* pMP::P*_mboII_* and the empty promoter-probe vector pMP220 was used as a control. The cultures were incubated at 22°C and 150 rpm, and samples were collected every twelve hours, until the stationary phase was reached. The optical density and the β-galatosidase activity were measured. The results are average of three independent experiments performed in triplicate. Error bars indicate standard deviation.

The 603 bp sequence corresponding to a non-coding region located upstream of *mboA* gene (Figure 4) was subjected to a promoter and transcription factor (TF) analysis using the BPROM programme. This region presented a significantly lower GC content than that in the entire *mbo* operon (48% versus 56%), suggesting the presence of a possible regulatory region [47]. Two predicted promoters were allocated upstream of the putative *mbo* operon, which were designated P*_mboI_* and P*_mboII_*. The *in silico* analysis of both putative promoters showed -10 and -35 boxes and TF binding sites (Figure 4). The first predicted promoter (P*_mboI_*) was located from position 74 to 120 (purple box), with a -10 box (TATTAGGAT) located at position 111, and the -35 box (TTGCAA) was located at position 91 (Figure 4). The *in silico* analysis also identified a putative TF binding site belonging to the *crp* binding sites with the sequence TTAGATTA at position 74 [48], [49]. The second predicted promoter (P*_mboII_*) was located at position 442 to 524 (green box), with a -10 box (TGTTGTGAT) located at position 515 and the -35 box (TTCAGG) was located at position 492 (Figure 4). In addition, a binding site for *rpoD17* was associated with this putative promoter at position 442. The activity produced by the combination of both putative promoters (P*_mbo_*), and the individual promoters (P*_mboI_* and P*_mboII_*, Figure 5A) was measured in PMS at 22°C in two different genetic backgrounds, the wild-type strain *P. syringae* pv. syringae UMAF0158 and the non-producing mangotoxin strain *P. syringae* pv. syringae B728a, which does not harbour any *mbo* homologous sequences. The results from the β-galactosidase activity assays demonstrated that P*_mboI_* is the primary active promoter for the production of mangotoxin in the conditions assayed (Figure 5B). The β-galactosidase activity levels for P*_mboII_* were null, which is similar to the results obtained with the empty vector pMP220 (Figure 5B). The β-galactosidase activity was clearly lower in *P. syringae* pv. syringae B728a as compared with the wild-type strain UMAF0158. When the promoters were cloned into the same construct in *P. syringae* pv. syringae B728a, the β-galactosidase activity reached 90 Miller units, and the pMP::P*_mboI_* reached 1,300 Miller units. However, the pMP::P*_mboII_* showed the same level of activity as that of the empty vector cloned into the wild-type strain (Figure 5B). In addition, the exact site of transcription in the *mbo* operon at the 5′ ends of the corresponding mRNA was determined using 5′RACE experiments. Three clones containing cDNA from the 5′-end of the transcript were randomly selected that had similar restriction patterns. The sequencing of these three clones revealed the precise site of transcription initiation (Figure 4). The transcription start site was located at position 142, which was 462 bp upstream from the *mboA* gene start codon. Remarkably, a comparison of the P*_mboI_* promoter sequence in different strains containing the orthologous sequence of the *mbo* operon showed a high level of conservation in the consensus DNA sequence for the -10 and -35 boxes and the *crp* binding site (Figure S4).

### Rho-independent Terminator is Allocated at the End of the *mbo* Operon

**Figure 6:**
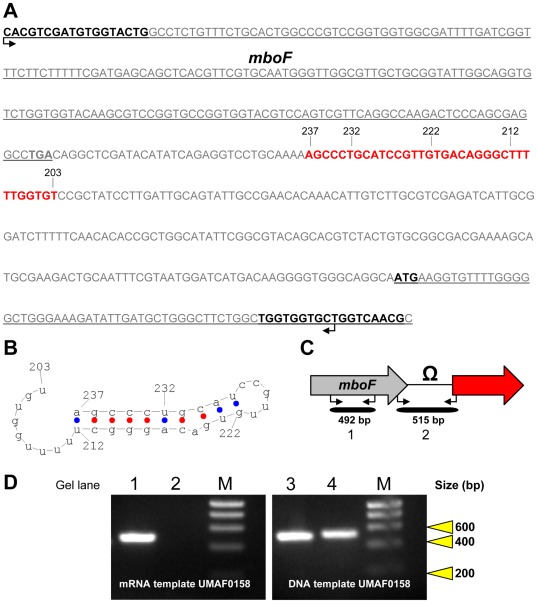
Analysis of the Rho-independent terminator located at the end of the *mbo* operon. (**A**) Nucleotide sequence of the terminal region of *mbo* operon. The 3′-end of *mboF* is underlined and the stop codon is shown in bold type. The 5′-end of the following gene, which is not involved in the production of mangotoxin, is also underlined and the start codon is shown in bold type. The location and sequence of the forward primer 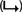 in the *mboF* gene and reverse primer 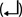 are also shown in bold type. The nucleotide sequence of the putative terminator located between these two genes is indicated in red letters. The numbers denote the nucleotide positions on the terminator sequence. (**B**) Secondary structure of the putative Rho-independent terminator of the *mbo* operon predicted using the FindTerm programme. The numbers denote the nucleotide positions on the terminator sequence. (**C**) Experimental design diagram to confirm the functional *mbo* operon terminator. The 3′ end organisation of the *mbo* operon showed *mboF* as the last gene in the operon. The amplicon sizes, the primers direction 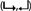 and the transcriptional terminator are indicated (Ω). (**D**) Agarose electrophoresis from the RT-PCR experiments in the wild-type strain according to the previous design (Figure 3 and Table S2); RT-PCR using mRNA: gel lines 1 (primer pair 13) and 2 (primer pair 14), and RT-PCR using genomic DNA: gel lines 3 (primer pair 13) and 4 (primer pair 14). HyperLadder I (Bioline) was used as a molecular size marker (M).

**Figure 7:**
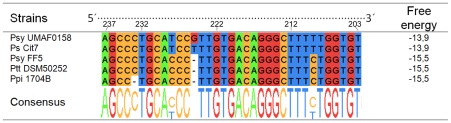
Comparison of Rho-independent terminator sequence motifs present in different strains of the *P. syringae* pathovars. This alignment was analysed using Jalview software. A summary of the tendency of each nucleotide to hold each position is represented under the alignment as a consensus sequence.

The non-coding sequence downstream of the *mboF* gene was analysed using the FindTerm programme to uncover putative a Rho-independent bacterial terminators (Figure 6A). The most likely candidate was a 30 bp sequence at position 32 downstream from the *mboF* stop codon (Figure 6A). This Rho-independent terminator sequence was analysed using the FoldRNA programme. This programme is used to predict secondary RNA structure through energy minimisation in order to calculate the free energy released during palindromic structure formation. The terminator sequences showed negative free energy values (−13.9 kcal mol^−1^ in 66% of helices), indicating that their formation would be favoured and spontaneous (Figure 6B). Finally, to confirm the functionality of the putative terminator, RT-PCR experiments were performed to amplify the 3′-end of the transcript using primers specific to sequences occurring before and after the putative terminator (Figure 6C). The RT-PCR analysis of the *mbo* transcript showed the absence of amplification in the non-coding region downstream of *mboF*, where the putative terminator was located, indicating that transcription is terminated before reaching the upcoming gene (Figure 6D). This sequence was the functional terminator of the *mbo* operon. Moreover, comparisons of the terminator sequences from the five available orthologous sequences of the *mbo* operon showed a high level of conservation of this terminator sequence in the different strains used in the experiment (Figure 7). Consistent with the bioinformatic analysis using FindTerm and FoldRNA, the terminator sequences of the strains analysed were able to form secondary structures compatible with a transcription terminator (Figure 7).

### The Bioinformatic Analysis Reveals the Specificity of the *mbo* Genes

**Figure 8:**
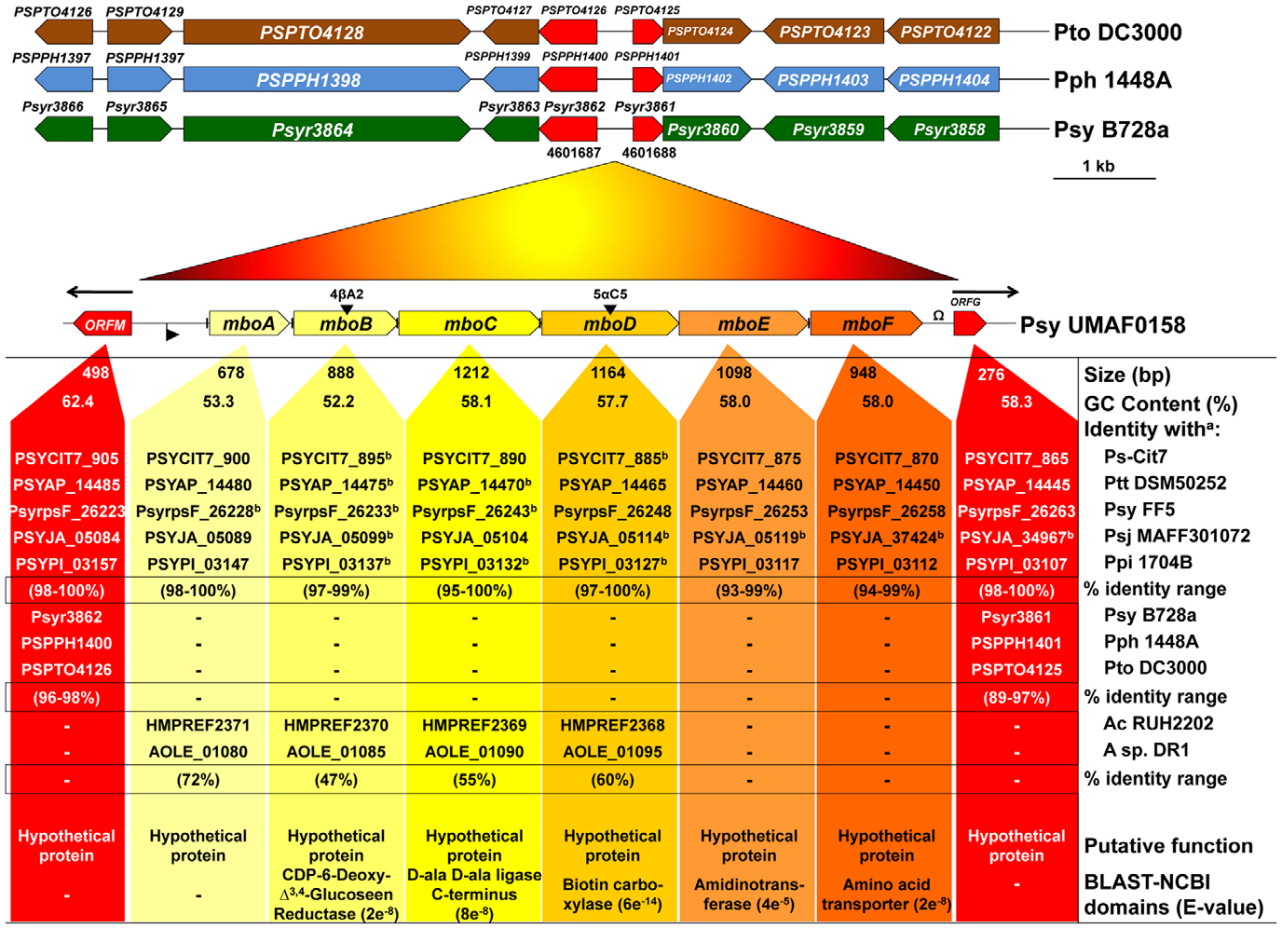
Bioinformatic analysis of the *mbo* operon. The flanking ORFs are shown in red. The sizes of each ORF, the GC content (%), the identity with other genes, the putative function and the presence of the domains with their corresponding E-values have been analysed. The position of the *mbo* operon in other *P. syringae* pathovars is also compared. Insertion sites of mini*Tn5* mutants are indicated (▾). The promoter located upstream of the *mbo A* gene (▸) and the transcription terminator downstream of the *mboF* gene (Ω) are also marked. Transcription directions of the flanking genes are indicated by arrows (←,→). The predicted SD sequences before each gene are indicated using a vertical line (―). The vertical arrows from yellow to orange bars exhibits also the percentages of identity with genes of other sequenced bacteria: *Acinetobacter* sp. (A) DR1, *Acinetobacter calcoaceticus* (Ac) RUH2202, *P. syringae* (Ps) Cit7, *P. syringae* pv. aptata (Ptt) DSM50252, *P. syringae* pv. japonica (Psj) MAFF301072, *P. syringae* pv. phaseolicola (Pph) 1448A, *P. syringae* pv. pisi (Ppi) 1704B, *P. syringae* pv. syringae (Psy) B728a and FF5 and *P. syringae* pv. tomato (Pto) DC3000. The absence of identity is indicated as - ^a^Identity at the amino acid level. ^b^Uncompleted sequences deposited in the databases.

Specialised BLAST-NCBI and Pfam databases were used for the specific bioinformatic analysis of each *mbo* gene (Figure 8). The six *mbo* genes encode hypothetical proteins with no identity to well-known proteins. However, each individual sequence was analysed to uncover predicted domains in the database. A putative Shine-Dalgarno sequence (SD) appeared upstream of *mboA* gene at nucleotide -9 (ATGGAGAG, Figure 4). The *mboA* gene encodes a conserved hypothetical protein, though no similarities with any of the conserved domains in the published protein sequences were found. The *mboB* gene contains a putative SD at -4 (AAGGTCGG), and homology protein domain searches for the MboB protein product revealed significant matches with a reductase protein at an E-value of 2e^−8^. The *mboC gene* showed significant matches with a D-ala D-ala ligase protein domain with an E-value of 8e^−8^ and harboured a putative SD (TCGGAGAC) at -6. The *mboD* gene containing a putative SD at -9 (ACAGAGGT) and the conserved-domain analysis of the amino acid derivate sequences showed similarities to biotin carboxylase with an E-value of 6e^−14^. The *mboE* gene contained amidinotransferase domains with E-values of 4e^−5^, but a putative SD site was not found. Finally, the *mboF* gene contains a putative SD site at position -6 (TTCGAGGG) and presents typical domains for amino acid transporters with an E-value of 2e^−8^. The entire *mbo* operon has been recently detected in five *P. syringae* draft genomes [50], with a high level of identity between 93 and 100%. The average GC content of the *mbo* operon (56%) was consistent with the GC content of *P. syringae* genomes [50], [51]. This *mbo* operon is not present in other *Pseudomonas* strains, including the completely sequenced and annotated *P. syringae* strains, such as B728a, 1448A and DC3000 (Figure 8). In addition, partial identity to the *mbo* operon (orthologous genes *mboA*, *B*, *C* and *D* with a low identity range (47–72%) has been detected in only two sequenced strains of *Acinetobacter*.

### Mangotoxin-producing Strains Show a Strong Phylogenetic Relationship

**Figure 9:**
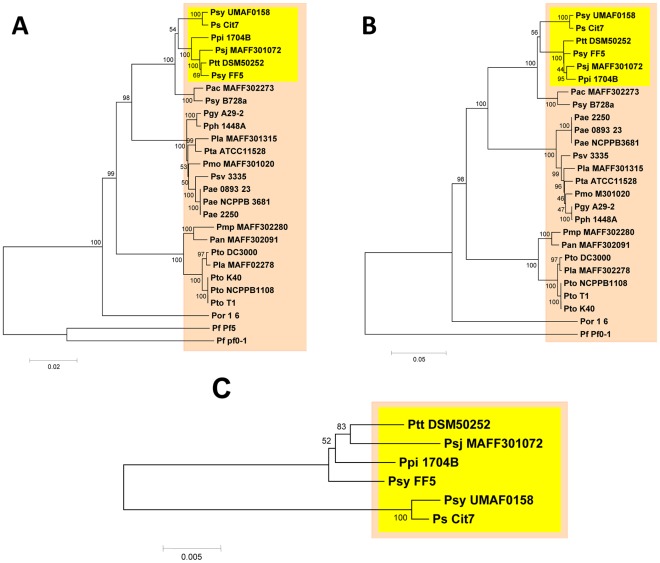
Phylogenetic analysis of the mangotoxin-producing and -non-producing *P. syringae* strains. Neighbour-joining trees were constructed using MEGA 4.0.2 bootstrap values (100,000 repetitions) are shown on branches. Abbreviations for *Pseudomonas* strains are given as: Pf, *fluorescens*; Psv, *P. savastanoi* pv. savastanoi; and for *P. syringae* pathovars are given as: Ps, *P. syringae* (no pathovar assigned); Pac, aceris; Pan, actinidae; Pae, aesculi; Ptt, aptata; Pgy, glycinea; Psj, japonica; Pla, lachrymans; Pmo, mori; Pmp, morsprunorum; Ppi, pisi; Pph, phaseolicola; Psy, syringae; Por, oryzae; Pta, tabaci; Pto, tomato. The tree was rooted with *P. fluorescens* Pf-5 and Pf0-1. Evolutionary distances are given in units of nucleotide substitutions per site. The topology was identical for trees produced by the minimum evolution and maximum parsimony methods. Sequences from all strains used were extracted from published genome sequences. Neighbour-joining trees were constructed using: (**A**) six concatenated genes (*fruK*, *gapA*, *gltA*, *pgi*, *recA* and *rpoD* genes), (**B**) the *mgo* operon concatenated genes (*mgoB*, *mgoC*, *mgoA and mgoD* genes), (**C**) the *mbo* operon concatenated genes (*mboA*, *mboB*, *mboC, mboD, mboE* and *mboF*) using only *P. syringae* strains where present.

Although no homology with orthologous genes in other strains was reported at the beginning of this work, the appearance of new sequences and completed genomes allowed the detection of homologies. Thus, these genes exhibited a high degree of identity with some of the genes present in the draft genome of five other strains recently sequenced from different pathovars of *P. syringae* (Table 3): Cit7, DSM50252, MAFF301072, 1704B [50] and FF5 (Pseudomonas syringae Genome Resources Home Page; http://www.pseudomonas-syringae.org, accessed on 10^th^ January 2012). The phylogenetic relationship of the *P. syringae* strains, with or without orthologous genes to the *mbo* operon, was analysed by comparing a set of six protein-coding housekeeping genes (*fruK, gapA, gltA, pgi, recA* and *rpoD,* Figure 9A). An additional phylogenetic analysis was performed by comparison of orthologous genes of the *mgo* operon (Figure 9B), which are involved in the production of mangotoxin [37], . Similarly, the phylogenetic relationship was analysed using only the orthologous genes to the *mbo* operon described in this study, using the six *P. syringae* strains where these genes have been found and sequenced (Figure 9C). This analysis showed that the housekeeping genes and the *mgo* operon were highly conserved among the twenty-seven strains examined, resulting in two phylogenetic diagrams that were similar (Figures 9A and B). The *mbo* operon was only found in six strains for which a draft genome has been recently published (Figure 9C). The phylogenetic relationship between these six *P. syringae* strains and other strains was determined using the *mgo* operon and housekeeping multilocus analyses (Figure 9). With respect to the organisation of the other strains used in this study, all *mbo* operon containing strains clustered into a single, clearly differentiated group.

**Table 3:** Description of strains and plasmids used in this study.

Strain/plasmid	Relevant characteristics^a^	Reference or source
**Strains**		
*E. coli*		
DH5α	*E. coli [F′ Φ80lacZ* Δ*M15* Δ*(lacZYA-argF)U169 deoR recA1 endA1 hsdR17* *(rK-mK+)phoA supE44 lambda- thi-1]*	[81]
CECT831	Indicator strain of production of mangotoxin	CECT^b^
*P. fluorescens*		
Pf-5	Complete genome, non mangotoxin producer, *mbo* operon absent	[46]
Pf0-1	Complete genome, non mangotoxin producer, *mbo* operon absent	[45]
*P. savastanoi* pv. savastanoi		
NCPPB3335	Draft genome, non mangotoxin producer, *mbo* operon absent	[51]
*P. syringae* Cit7	Draft genome, possess *mbo* operon	[50]
*P. syringae* pv. aceris		
MAFF302273	Draft genome, *mbo* operon absent	[50]
*P. syringae* pv. actinidae		
MAFF302091	Draft genome, *mbo* operon absent	[50]
*P. syringae* pv. aesculi		
0893_23	Draft genome, *mbo* operon absent	[50]
2250	Draft genome, *mbo* operon absent	PPI web page (D. Studholme)
NCPPB3681	Draft genome, *mbo* operon absent	PPI web page (D. Studholme)
*P. syringae* pv. aptata		
DSM50252	Draft genome, possess *mbo* operon	[50]
*P. syringae* pv. glycinea		
A29-2	Draft genome, *mbo* operon absent	[50]
*P. syringae* pv. japonica		
MAFF301072	Draft genome, possess *mbo* operon	[50]
*P. syringae* pv. lachrymans		
MAFF301315	Draft genome, *mbo* operon absent	[50]
MAFF302278	Draft genome, *mbo* operon absent	[50]
*P. syringae* pv. mori		
MAFF301020	Draft genome, *mbo* operon absent	[50]
*P. syringae* pv. morsprunorum		
MAFF302280	Draft genome, *mbo* operon absent	[50]
*P. syringae* pv. oryzae		
1_6	Draft genome, *mbo* operon absent	[50]
*P. syringae* pv. phaseolicola		
1448A	Complete genome, non mangotoxin producer, *mbo* operon absent	[54]
*P. syringae* pv. pisi		
1704B	Draft genome, possess *mbo* operon	[50]
*P. syringae* pv. syringae		
B728a	Complete genome, non mangotoxin producer, *mbo* operon absent	[52]
FF5	Draft genome, non mangotoxin production detected, possess *mbo* operon	PPI web page (D. Studholme)
UMAF0158	Wild type, isolated from mango, mangotoxin producer, Nf^r^	[39]
UMAF0158-4βA2	mini*Tn5* mutant of UMAF0158 in *mboB* defective in mangotoxin, Km^r^, Nf^r^	[37]
UMAF0158-5αC5	mini*Tn5* mutant of UMAF0158 in *mboD* defective in mangotoxin, Km^r^, Nf^r^	[37]
UMAF0158-2βB7	mini*Tn5* mutant of UMAF0158 in *gacA* defective in mangotoxin, Km^r^, Nf^r^	[37]
*mgoA* ***^−^***	*mgoA* mutant of UMAF0158 by deletion, Nf^r^	[42]
*mboA* ***^−^***	*mboA* mutant of UMAF0158, *mboA*:: pCR*::mboA*, Km^r^, Nf^r^	This study
*mboB* ***^−^***	*mboB* mutant of UMAF0158, *mboB*:: pCR::*mboB*, Km^r^, Nf^r^	This study
*mboC* ***^−^***	*mboC* mutant of UMAF0158, *mboC*:: pCR::*mboC*, Km^r^, Nf^r^	This study
*mboD* ***^−^***	*mboD* mutant of UMAF0158, *mboD*:: pCR::*mboD*, Km^r^, Nf^r^	This study
*mboE* ***^−^***	*mboE* mutant of UMAF0158, *mboE*:: pCR::*mboE*, Km^r^, Nf^r^	This study
*mboF* ***^−^***	*mboF* mutant of UMAF0158, *mboF*:: pCR::*mboF*, Km^r^, Nf^r^	This study
*P. syringae* pv. tabaci		
ATCC11528	Draft genome, *mbo* operon absent	[50]
*P. syringae* pv. tomato		
DC3000	Complete genome, non mangotoxin producer, *mbo* operon absent	[25]
PT23	Non mangotoxin producer, *mbo* operon absent	[27]
T1	Draft genome, *mbo* operon absent	[53]
NCPPB1108	Draft genome, *mbo* operon absent	PPI web page (B. Vinatzer)
K40	Draft genome, *mbo* operon absent	PPI web page (B. Vinatzer)
**Plasmids**		
pBBR1MCS-5	Gm^r^; 4.7 kb broad-host-range cloning vector	[44]
pCG1-5	UMAF0158 genomic DNA (12,509 bp) cloned in pBlueSTAR-1	This study
pCR2.1	Ap^r^, Km^r^; 3.9 kb cloning vector	Invitrogen, California, USA
pCR::*mboA*	integrative plasmid pCR2.1 carrying a *mboA* fragment	This study
pCR::*mboB*	integrative plasmid pCR2.1 carrying a *mboB* fragment	This study
pCR::*mboC*	integrative plasmid pCR2.1 carrying a *mboC* fragment	This study
pCR::*mboD*	integrative plasmid pCR2.1 carrying a *mboD* fragment	This study
pCR::*mboE*	integrative plasmid pCR2.1 carrying a *mboE* fragment	This study
pCR::*mboF*	integrative plasmid pCR2.1 carrying a *mboF* fragment	This study
pGEM-T	Ap^r^; 3.0 kb cloning vector	Invitrogen, California, USA
pGEM-T AF	*mboABCDEF* cloned in pGEM-T	This study
pLac-AF	*mboABCDEF* cloned in pBBR1MCS-5 in the same direction than the *lacZ* promoter in the vector	This study
pLac-FA	*mboABCDEF* cloned in pBBR1MCS-5 in the opposite direction of the constitutive promoter in the vector	This study
pMP220	Promoter-probe vector containing a promoterless LacZ gene	[84]
pMP::P*_mbo_*	pMP220 vector containing the two putative promoters of P*_mbo_* operon (603 bp)	This study
pMP::P*_mboI_*	pMP220 vector containing the first putative promoter of *mbo* operon (294 bp)	This study
pMP::P*_mboII_*	pMP220 vector containing the second putative promoter of *mbo* operon (360 bp)	This study

CECT: Spanish Type Culture Collection; NCPPB: National Collection of Plant Pathogenic Bacteria, Harpenden, UK; MAFF: Ministry of Agticulture, Forestry and Fisheries, Tsukuba, Ibaraki, Japan; DSM: DSMZ-Deutsche Sammlung von Mikroorganismen und Zellkulturen GmbH, Braunschweig, Germany.

a Amp^r^: ampicillin resistance; Gm^r^: gentamicin resistance; Km^r^: kanamycin resistance; Nf^r^: Nitrofurantoin resistance.

## Discussion

Mangotoxin is a virulence factor that contributes to *P. syringae* pv. syringae fitness and host interactions [6]. Mangotoxin is produced in several pathovars of *P. syringae*. Murillo and co-workers have demonstrated that the strain *P. syringae* pv. syringae CFBP 3388 produces mangotoxin and phaseolotoxin and the pv. avellanae produces mangotoxin [41]. In our group, mangotoxin production in the pv. pisi has been demonstrated (V.J. Carrión, unpublished data). Recently sequenced strains have revealed the presence of the *mbo* operon in the pathovars aptata, japonica and pisi [50]. In this work, the phytopathogenic strain *P. syringae* pv. syringae UMAF0158 was used to characterise the genetic basis of mangotoxin production. The aim of this study was to describe and characterise the *mbo* operon (*mangotoxin biosynthetic operon*), which is essential for the production of mangotoxin.

A 12,509 bp region from the *P. syringae* pv. syringae UMAF0158 chromosome, which includes thirteen ORFs, has been sequenced. Six ORFs were specific to mangotoxin-producing strains and were involved in the production of mangotoxin. These six ORFs formed the denominated *mbo* operon. A comparison of the *mbo* operon sequence to the corresponding chromosome region of some sequenced *Pseudomonas* resulted in a correlation between the presence of these six genes and the production of mangotoxin [41]. The absence of these *mboABCDEF* genes in the genome of mangotoxin non-producing *P. syringae* (as pv. syringae B728a, pv. phaseolicola 1448A and pv. tomato DC3000) is consistent with our results. It is known that the genes for the biosynthesis of some phytotoxins, such as coronatine and phaseolotoxin, have been lost in the genomes of some *P. syringae* strains [52], [53], [54].

The insertional mutants could generate polar effects in the genes allocated downstream the insertion. This could result in a failure in the transcription of these genes downstream. In our case, those polar effects were absent, as it was shown by the RT-PCR analysis. The absence of polar effects could be due to the strong activity of the P*_mboI_* promoter. Further evidence that the genes forming the *mbo* operon are involved in the production of mangotoxin is provided by the results of the disruption of *mboA* to *mboD*, in which no mangotoxin production was detected (Tox^-^ phenotypes) in the antimetabolite toxin detection bioassay. The insertional mutants in *mboE* and *mboF* showed an alteration, but not absence, of the production of mangotoxin. These data indicate that the six genes within the *mbo* operon encode proteins required or involved at any of the different stages of mangotoxin production, such as synthesis, transport, and/or regulation, in a *Pseudomonas* genetic background. Curiously, the six *mbo* insertional and mini*Tn5* random mutants showed residual toxic activity from an uncharacterised toxic compound with a different target, which was not reversed in the presence of ornithine, an amino acid that restores the mangotoxin action in the antimetabolite toxin detection bioassay.

To assign functionality to the *mbo* genes, complementation experiments were designed. For the mutant complementation, we constructed pLac-AF and pLac-FA plasmids, which contain the *mbo* operon cloned in both directions, to generate transcripts under the constitutive promoter of the vector (P*_LAC_*) or their own expression promoter (P*_mboI_*). The mangotoxin production in all *mbo* mutants was restored when they were transformed with these plasmids. Moreover, when mangotoxin non-producing *P. syringae* strains were transformed with the pLac-AF or pLac-FA plasmids, most of these strains were able to produce mangotoxin. One interesting exception is the *P. syringae* pv. syringae FF5 strain. This strain possessed orthologous genes to the *mbo* and *mgo* operons, but it did not produce mangotoxin. However, FF5 is able to undergo mangotoxin production when it is transformed with the pLac-AF or pLac-FA plasmids. After the comparison of both *mbo* operon sequences (data not shown), the number of specific nucleotide changes suggests that FF5 might have specific mutations and/or alterations in this operon that could result in the generation of a mangotoxin non-producing strain. Similar results have been reported for the synthesis of the lipopeptide surfactin in the Gram-positive *Bacillus subtilis* [55]. Other exceptions were detected in the *P. fluorescens* strains Pf0-1 and Pf5. The non-producing strains Pf0-1 and Pf5 synthesised mangotoxin only in the presence of the pLac-AF vector, which drives the expression of mangotoxin under the control of a constitutive promoter. This phenotype could be associated with the modification or absence of the important *mgo* genes, which were previously reported for the production of mangotoxin. In fact, the *mgoA* gene encodes a non-ribosomal peptide synthetase that is involved in the production of mangotoxin [37], [42]. Our data suggest that *mgoA* could be a specific regulator for the expression of these genes, as it can be observed in Figure 3C. Recently, orthologous *pvf* genes have been detected in *P. enthomophila*, which were related with the regulation of virulence factor in *Pseudomonas* spp. [43]. The orthologous *mgoA* gene of Pf0-1 showed a low identity (63%), suggesting the presence of changes in the MgoA protein structure. However, Pf-5 did not contain orthologous *mgo* genes. These mangotoxin-non-producing strains changed their phenotypes to mangotoxin producers when they were transformed with the *mbo* operon, suggesting that the *mbo* operon is essential and specific for the production of mangotoxin.

The organisation of the *mbo* operon sequence was consistent with the characteristics of an operon as previously described, with genes separated by less than 20–30 bp [56], [57], [58]. In addition, the presence of the SD sequence supports the existence of an *mbo* operon [57], [59]. Moreover, the *mbo* genes have been reported to be co-transcribed in a unique polycistronic transcriptional unit, flanked by an active promoter and a Rho-independent transcriptional terminator. The site of transcription initiation for the *mbo* operon was located at 462 bp upstream from the *mboA* gene start codon. This distance is consistent with the promoters described in *P. syringae* [60]. The predicted -10 and -35 boxes were highly conserved in the orthologous *mbo* operon and present in other *P. syringae* strains. Additionally, a TF *crp* binding site was located in close proximity to the promoter and a putative Crp protein in the genome of *P. syringae* pv. syringae UMAF0158. Consistent with the published data concerning virulence factors in *P. aeruginosa*, the Vfr protein, which is homologous to the Crp protein, is involved in the control of the type III secretion system, quorum-sensing response and several virulence factors [61], [62], [63], suggesting that mangotoxin production could be under similar control.

The sequence analysis of *mbo* genes disrupted in the non-producing mangotoxin mutants allowed us to assign predicted functions in mangotoxin production. The domain analysis of the *mboA* gene did not generate any results from the databases; however, this protein must play an essential role in the biosynthesis of mangotoxin because its disruption inhibits mangotoxin production. The disruption of the *mboB* gene also inhibited mangotoxin production. The domain analysis of the *mboB* gene revealed domains consistent with a CDP-6-deoxy- Δ^3,4^-glucoseen reductase [64]. This function is generally related with oxidoreductive processes in the metabolism of the 3,6-dideoxyhexoses in the lipopolysaccharides of Gram-negative bacteria and could also serve as electron donors during mangotoxin biosynthesis [65], [66]. The *mboC* gene presents a domain consistent with the D-ala D-ala ligase C-terminus. This enzyme is used to catalyse the interaction of the carboxylic terminus with an amine group using ATP [67], [68], [69]. In the case of peptide bond synthesis, nonribosomal multi-enzyme complexes are used in the stepwise transfer of a phosphoryl group to a carboxylate, yielding aminoacyl phosphate intermediates during mangotoxin biosynthesis [37], [67]. The involvement of D-ala-D-ala domain proteins has also been described in other antimetabolite toxins, such as the protein PSPPH_4299, which functions in phaseolotoxin biosynthesis [70]. Recently obtained unpublished preliminary data suggest that the mangotoxin molecule could comprise a dipeptide (Dr. D. Romero, personal communication), with compatible D-ala D-ala ligase activity in the *mboC* gene. The MboD protein presents a biotin carboxylase domain. No homologous genes were detected in other antimetabolite toxin-producing bacteria that might help to elucidate the role of this gene in the production of mangotoxin. The biotin carboxylase function is related to the integration of the carbon atom in a molecule with a considerable energy cost [69], [71]. The *mboE* gene encodes a protein with an amidinotransferase domain. The gene *amtA* in *P. syringae* pv. phaseolicola NPS3121 is an amidinotransferase involved in the formation of homoarginine, which is a component of the chemical structure of phaseolotoxin [28]. Therefore, *mboE* could possess a similar function to produce changes in the partially active toxin to transform it into the completely active mangotoxin, which could explain the halo that is present in its insertional mutant. Finally, the protein encoded by the *mboF* gene has two domains of EamA family, which belongs to the DMT superfamily of drug/metabolite transporters [72]. These proteins are involved in the transport of amino acids, purines and other metabolites outside the cell [73]. The YdeD transporter, which is present in EamA domains in *E. coli*, has been extensively studied and is involved in the removal of metabolites of the cysteine biosynthesis pathway [73], [74], [75].

Finally, to gain insight concerning the phylogeny among different *Pseudomonas* spp. strains, we performed phylogenetic studies using the housekeeping genes *mgo* and *mbo*. Recent studies have revealed that genes orthologous to the *mgo* operon that are involved in the production of mangotoxin are present in the majority of species and pathovars of *Pseudomonas* [43], [76]. When the *mgo* genes were used, the phylogenetic analysis showed a similar organisation, displaying one grouping of the six strains, which harbour the *mbo* genes. The results of the cluster analysis are consistent with previous phylogenetic studies on *P. syringae* strains; thus, all strains used in this study belong to the genomospecies [77], [78], suggesting that the *mbo* operon was acquired one time during the evolution. Moreover, our results show that the strains that possess the *mbo* operon are limited to a branch of evolution presenting a common ancestor. Additionally, a comparative study among the six strains using the *mbo* operon has revealed two related groups of *P. syringae*, suggesting a slight variability among the studied sequences.

The characterisation of the *mbo* operon reveals the existence of a new group of genes involved in the production of mangotoxin in *P. syringae* strains. These *mbo* genes are specifically detected in *P. syringae* strains and confer the ability to produce mangotoxin in mangotoxin non-producing strains. In other *Pseudomonas* spp., the production of mangotoxin was also detected, but only when this operon is under constitutive expression, revealing the genetic background importance of the producer strain. Typical features of an operon have been described for the *mbo* genes, such as the presence of an active promoter, a Rho-independent terminator or unique polycistronic mRNA transcription. In this study, we confirmed the role of *mbo* genes in the production of mangotoxin. The phylogenetic studies reveal a strong relationship among *mbo*-harbouring *Pseudomonas* strains.

## Methods

### Bacterial Strains and Plasmids Used in this Study

*P. syringae strains* were grown at 22°C in King’s B medium (KMB) [79]. The plasmids were introduced into *Pseudomonas* strains by electroporation using a Gene Pulser Xcell System (Bio-Rad Laboratories) according to the manufacturer’s instructions. The electrocompetent cells were obtained according to the method of Choi *et al.* [80]. *Escherichia coli* DH5α [81] were grown in Luria–Bertani medium (LB) at 37°C, and transformation was accomplished by introducing plasmid DNA into competent cells using a standard protocol [82]. The media was supplemented with the appropriate antibiotics when necessary (ampicillin, 100 µg ml^−1^; streptomycin, 50 µg ml^−1^; tetracycline, 20 µg ml^−1^; kanamycin, 50 µg ml^−1^; and gentamicin, 30 µg ml^−1^).

### Detection of *P. syringae* Toxins Production

The mangotoxin production was assayed using the indicator technique, which has been previously described [83] and involves growth inhibition of *E. coli* on Pseudomonas minimal medium (PMS). Briefly, a double layer of the indicator microorganism was generated using the *E. coli* strain CECT831. After solidification, the *P. syringae* wild-type strain and its derivatives mutants were stabbed into the agar plates and incubated at 22°C for 24 h followed by an additional 24 h incubation at 37°C. To confirm the targeting of mangotoxin, 100 µl of a 100 mM solution of ornithine or N-acetyl-ornithine was assayed on individual plates. To assess the production of mangotoxin in the liquid cultures, we performed at cell-free filtrate dilution as previously described [40].

### Construction of UMAF0158 Mutants and Derivative Strains

The insertional inactivation mutagenesis of *P. syringae* pv. syringae UMAF0158 was used to suppress the production of mangotoxin by inserting disruption vectors into the different ORFs of the *mbo* operon by single-crossover homologous recombination. To construct the integrative plasmids (Table 3), DNA fragments from the different ORFs within the gene cluster were obtained using PCR with primers (Table S1) specific to the sequence of the genomic clone pCG1-5 (JQ409468). The PCR, cloning and plasmid purification were performed following standard procedures. The plasmids were transformed into the wild-type strain UMAF0158 by standard electroporation [80]. The mangotoxin-deficient phenotype of the mutants was evaluated using the mangotoxin assay described above. Additionally, the mutants were analysed using PCR and Southern blot analyses with the antibiotic resistance cassette or partial target gene sequences as probes to confirm gene disruption and select single-copy transformants.

### Complementation Experiments

A plasmid containing the six ORFs and regulatory sequences was constructed for complementation of the mutants in the *mbo* operon. A fragment containing *mboABCDEF* (7,086 bp) was amplified using PCR from UMAF0158 with primers SBC-AFHindIIIfor (5′- aggcAAGCTTgcgcatagcgatcg -3′) and SBC-AFXbaIrev (5′- cgcTCTAGAgaccagcaccaccag -3′), which added two additional restriction sites, *Hind*III to the 5′-end and *Xba*I in 3′-end. The amplification was conducted using a high-fidelity Taq polymerase (Expand Long Range, dNTPack, Roche), and the PCR product was cloned into the pGEM-T vector (Invitrogen). Subsequently, the DNA was removed from the vector by digestion with *Hind*III and *Xba*I and cloned into the *Hind*III/*Xba*I site of pBBR1MCS-5 [44], to produce the plasmid pLac-AF. The DNA fragment was fused downstream from the *lacZ* promoter in pBBR1MCS-5, where the polylinker is located. The *lacZ* promoter acts as constitutive promoter in *P. syringae*. However, we also cloned the DNA fragment in opposition to the direction of the polylinker to produce pLac-FA, where the putative *mbo* operon is active with its own promoter (Figure S3). The complementing plasmids (pLac-AF and pLac-FA) were introduced into *mbo* mutants and other mangotoxin non-producing *Pseudomonas* spp. using standard electroporation (Table 2).

### RNA Extraction and Northern Blot Analysis

RNA was isolated from cultures of *P. syringae* pv. syringae UMAF0158 grown for 48 h at 28°C in KMB agar to prepare a bacterial suspension in PMS minimal medium with a final optical density of 1.0 at 600 nm (approximately 10^9^ cfu ml^−1^). One millilitre of this suspension was used to inoculate 100 ml of PMS minimal medium. The bacteria were incubated at 22°C for 48 h with orbital shaking. Total RNA was extracted from cells using TRIzol reagent as recommended by the manufacturer (Invitrogen). The isolation of RNA from the bacterial culture was performed using the commercial kit NucleoSpin RNA Plant (Macherey-Nagel). The RNA concentration was determined using a Nanodrop ND-1000. The integrity of the RNA sample was assessed by agarose gel electrophoresis. Northern blot was performed using a denaturing agarose gel (0.7%) and formaldehyde (2.2 M). The samples were prepared with 30 µg of total RNA in MOPS running buffer with 2.2 M formaldehyde and 50% formamide, with denaturing at 65°C for 10 min. The RNA samples were run for 2 h at 60 V and were transferred to the nylon membrane by capillary diffusion using 10× SSC. The RNA samples were immobilised by UV cross-linking. The hybridisation was performed using probes labelled with DIG according to the manufacturer’s instructions (Roche).

### Reverse Transcription-PCR Analysis

DNA-free RNA was obtained from cultures grown in PMS broth for 48 h at 22°C. The RNA concentration was determined using a Nanodrop ND-1000 and was optimised to 50 ng µl^−1^ The RNA integrity was confirmed by agarose gel electrophoresis and then used for reverse transcription (RT). RT-PCR was performed using the Titan OneTube RT-PCR system with 100 ng of RNA in a final reaction volume of 50 µl according to the manufacturer’s instructions (Roche). The primers were designed using sequences located between and within each *mbo* gene (Figure 3A and Table S2). The RT reaction was performed at 50°C for 40 min, followed by PCR amplification using a 40-cycle amplification programme (94°C for 30 s, 58°C for 1 min, and 68°C for 1 min) and a final extension cycle at 68°C for 7 min. Positive control reactions containing DNA isolated from each corresponding bacterial strain were included in all assays.

### Characterization of *mbo* Operon Promoter

The bioinformatics analysis of the *mbo* operon suggested the presence of two putative promoters localised upstream of the *mboA*. The putative promoter regions were cloned in combination and separately into pMP220 using the β-galactosidase gene as a marker of putative promoter activities [84]. The cloning resulted in three constructs (Figure 5A and Table 3): 1) pMP::P*_mbo_*, which contains the both putative promoters; 2) pMP::P*_mboI_*, which contains the first putative promoter detected; and 3) pMP::P*_mboII_*, which contains the second putative promoter detected. The amplicons were cloned into pMP220 using the restriction enzymes *Eco*RI and *Pst*I. The resulting plasmids were transformed into *P. syringae* pv. syringae strains UMAF0158 *and* B728a (Figure 5 and Table 3) for the β-galactosidase assays using the protocol described by Miller (1972) with minor changes [85]. Briefly, an overnight culture (10 ml) of the *Pseudomonas* strains was grown for 48 h at 28°C in LB to prepare a bacterial suspension with an optical density of 1.0 at 600 nm (approximately 10^9^ cfu ml^−1^). One millilitre from this bacterial suspension was used to inoculate 100 ml of PMS minimal medium. The culture was incubated at 22°C until the stationary phase under orbital shaking. The samples were collected every 6 or 12 hours, and the cells were harvested and suspended in assay buffer to eliminate any error in the detection of β-galactosidase enzyme activity due to the effects of different carbon sources present in the growth medium. The results presented are all from three experiments, which were conducted in triplicate. To eliminate read-through activity from other promoters on pMP220, bacteria carrying this plasmid were utilised as the negative control.

### Mapping the site of Transcription Initiation

The transcription start point for the *mbo* operon was determined using the 5′ cRACE method [86], [87], [88]. The synthesis of single-stranded cDNA was performed using Total DNA-free RNA, which was obtained from cultures grown in PMS medium for 48 h at 22°C. One microgram of this RNA was used as a template to synthesize the first-strand cDNA by using a cDNA synthesis kit (SMART™ RACE cDNA Amplification Kit, Clontech), a gene-specific oligonucleotide primer designed to anneal within the coding region of the gene. The reactions proceeded for 90 min at 42°C. Then they were diluted in water 10-fold, and 1 µl of these dilutions was put into 20 µl of PCR mixture. The cycling profile was: 5 cycles for 30 s at 94°C; 3 min at 72°C; 5 cycles for 30 s at 94°C; 30 s at 70°C; 3 min 72°C; 25 cycles for 30 s at 94°C; 30 s at 68°C; 3 min at 72°C. The amplification products were cloned into the vector pGEM®-T Easy Vector (Promega Corporation) and sequenced.

### Phylogenetic Analysis

The phylogenetic analysis of *P. syringae* pv. syringae UMAF0158 and other strains belonging to the genus *Pseudomonas* was performed using multilocus sequence analysis and a concatenated data set of *fruK*, *gapA*, *gltA*, *pgi*, *recA*, *rpoD* and *gyrB* genes (partial sequences). Multiple alignments were performed with ClustalW [89], and a phylogenetic tree was obtained using the neighbour-joining method [90]. The percentage of replicate trees in which the associated taxa were clustered in the bootstrap test (100,000 replicates) was shown next to the branches [91]. All positions containing gaps and missing data were eliminated from the dataset (complete deletion option). The concatenated sequences of the housekeeping genes yielded an alignment with 9796 sites that could be compared among all strains. The phylogenetic analyses were conducted using MEGA 4.0.2 [92]. The phylogeny was also reported for the *mgo* (4233 sites) and *mbo* operons (4152 sites).

### Bioinformatics Analysis

Database searches were performed using the National Centre for Biotechnology Information website. Searches for sequence similarity in the NCBI databases and the analysis of conserved protein domains were performed using BLAST algorithms [93], protein tools from the EMBL European Bioinformatics Institute (http://www.ebi.ac.uk) and the Pfam database. Restriction maps were constructed and analysed using the JustBio website (http://www.jusbio.com). The primers were designed using Primer3 online software (http://primer3.sourceforge.net). Genome and nucleotide sequences were visualised and manipulated using the Artemis genome browser [94] and compared using ACT [95] in combination with WebACT [96]. The plasmid maps were constructed using the programme Plasmid Map Enhancer 3.1 (Scientific and Educational Software). The promoter (BPROM) and terminator (FindTerm and FoldRNA) prediction was performed using SoftBerry online programmes (http://www.softberry.com, Mount Kisco, NY, USA). The SD sequences have been defined according to Ma *et al*. [57]. The sequences GGAG, GAGG, and AGGA were searched manually to identify core SD motifs [57].

## Supporting Information

Figure S1 **Pairwise alignments between the genome of** ***P. syringae*** **pv. syringae B728a and pCG1-5 from** ***P. syringae*** **pv. syringae UMAF0158 (Psy B728a and Psy UMAF0158 pCG1-5).** Axes represent the genes in the order in which they occur on the chromosomes. Top axis, pCG1-5; bottom axis, B728a. The co-linear regions of similarity on both genomes are represented in red. The same alignments were also performed with pCG1-5 from *P. syringae* pv. syringae UMAF0158, *P. syringae* pv. phaseolicola 1448A and *P. syringae* pv. tomato DC3000 with similar results. The display was generated using the Artemis comparison tool (ACT, http://www.sanger.ac.uk/software/artemis/ACT).
(TIF)

Figure S2 **Polarity determination of insertional mutants by RT-PCR experiments.** RT-PCRs of the internal and intergenic regions were performed with RNA obtained from different insertional *P. syringae* pv. syringae UMAF0158 mutants in each gene of *mbo* operon. The primer pairs used for each reaction are detailed in Table S2 and schematic representation of the amplification fragments is showed in the Figure 3A. PCR performed with the same primer pairs, with RNA isolated from the wild-type strain and with genomic DNA as a positive control are also shown in Figure 3B.
(TIF)

Figure S3 **Construction of the pLac-AF and pLac-FA vector derivatives from pBBR1MCS-5.** The complete mbo operon, including the regulatory sequences (putative promoter and terminator), was cloned into both vectors. In pLac-AF, the *mbo* operon is under the control of the P*_LAC_* promoter with constitutive expression in *Pseudomonas* spp. and the own promoter of the mbo operon, whereas the pLac-FA vector is affected by only the own endogenous *mbo* operon promoter.
(TIF)

Figure S4 **Comparison of the P** ***_mboI_*** **sequence motif between different** ***P. syringae*** **strains.** This alignment was analysed using Jalview software. A summary of the tendency of each nucleotide to hold each position is represented under the alignment as a consensus sequence. The predicted -10 (position 21), -35 (position 42) boxes and *crp* box are marked in solid line.
(TIF)

Table S1 **Primers used in** ***mbo*** **genes mutation experiments, amplicons containing an internal fragment of each gene, were cloned in pCR2.1 for mutagenesis by integration.**
(DOC)

Table S2 **Primers used in RT-PCR experiments. NC1 and NC2 correspond to non-coding adjacent region upstream to** ***mboA*** **gene.**
(DOC)
