# Supplementary figures and images for: The mbo Operon Is Specific and Essential for Biosynthesis of Mangotoxin in Pseudomonas syringae
Source: PLoS One. 2012 May 17;7(5):e36709. doi: 10.1371/journal.pone.0036709 (PMC3355146; doi:10.1371/journal.pone.0036709)

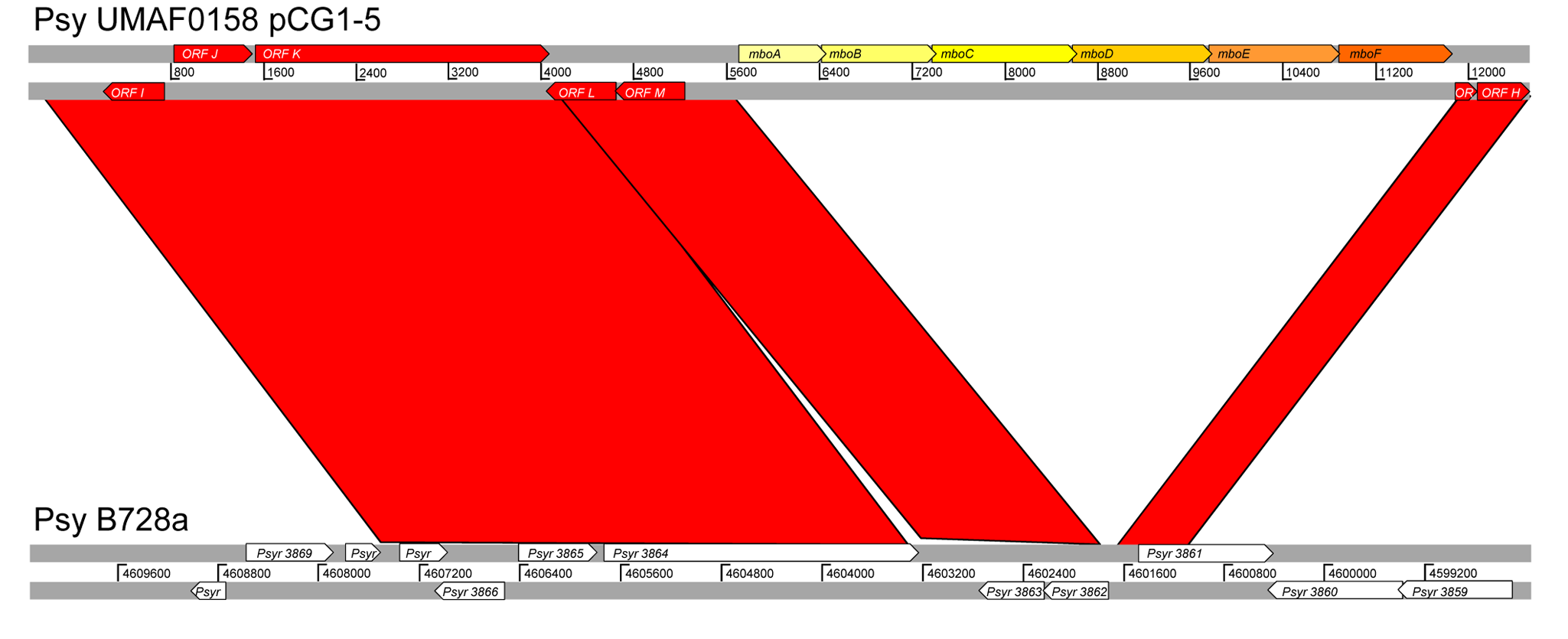

Supplement: Figure S1 — Pairwise alignments between the genome of P. syringae pv. syringae B728a and pCG1-5 from P. syringae pv. syringae UMAF0158 (Psy B728a and Psy UMAF0158 pCG1-5). Axes represent the genes in the order in which they occur on the chromosomes. Top axis, pCG1-5; bottom axis, B728a. The co-linear regions of similarity on both genomes are represented in red. The same alignments were also performed with pCG1-5 from P. syringae pv. syringae UMAF0158, P. syringae pv. phaseolicola 1448A and P. syringae pv. tomato DC3000 with similar results. The display was generated using the Artemis comparison tool (ACT, http://www.sanger.ac.uk/software/artemis/ACT). (TIF) [file pone.0036709.s001.tif]

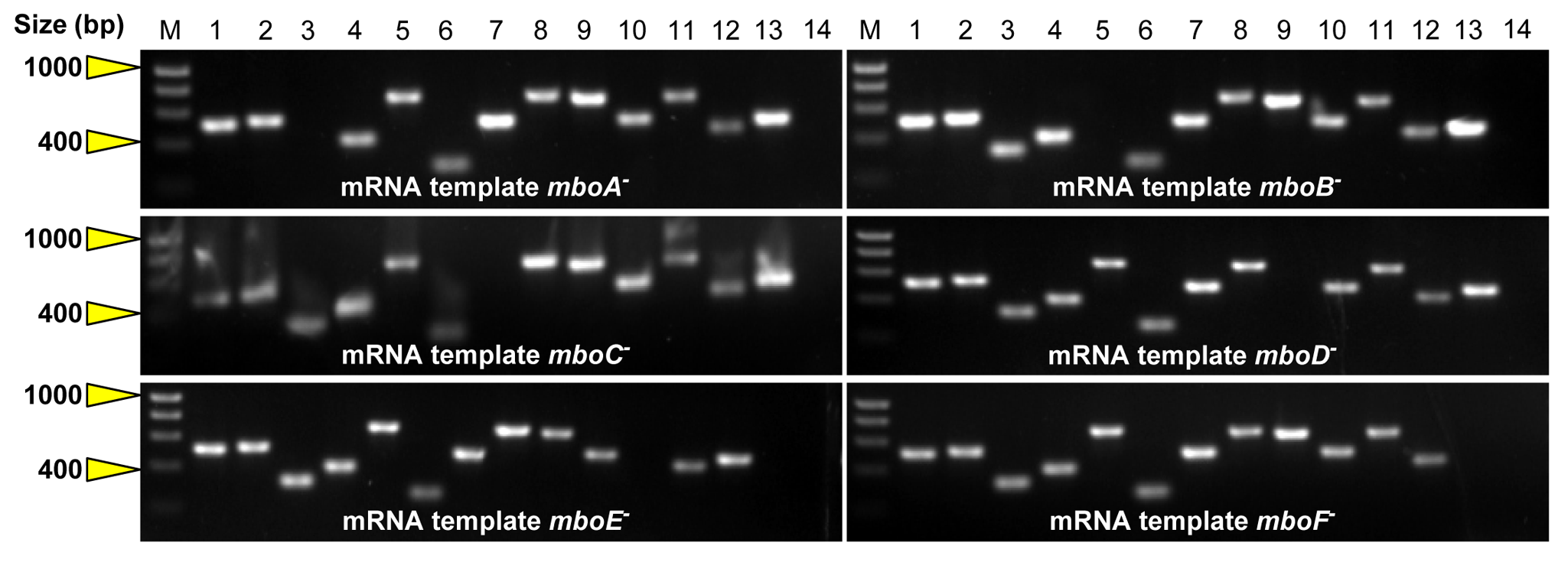

Supplement: Figure S2 — Polarity determination of insertional mutants by RT-PCR experiments. RT-PCRs of the internal and intergenic regions were performed with RNA obtained from different insertional P. syringae pv. syringae UMAF0158 mutants in each gene of mbo operon. The primer pairs used for each reaction are detailed in Table S2 and schematic representation of the amplification fragments is showed in the Figure 3A. PCR performed with the same primer pairs, with RNA isolated from the wild-type strain and with genomic DNA as a positive control are also shown in Figure 3B. (TIF) [file pone.0036709.s002.tif]

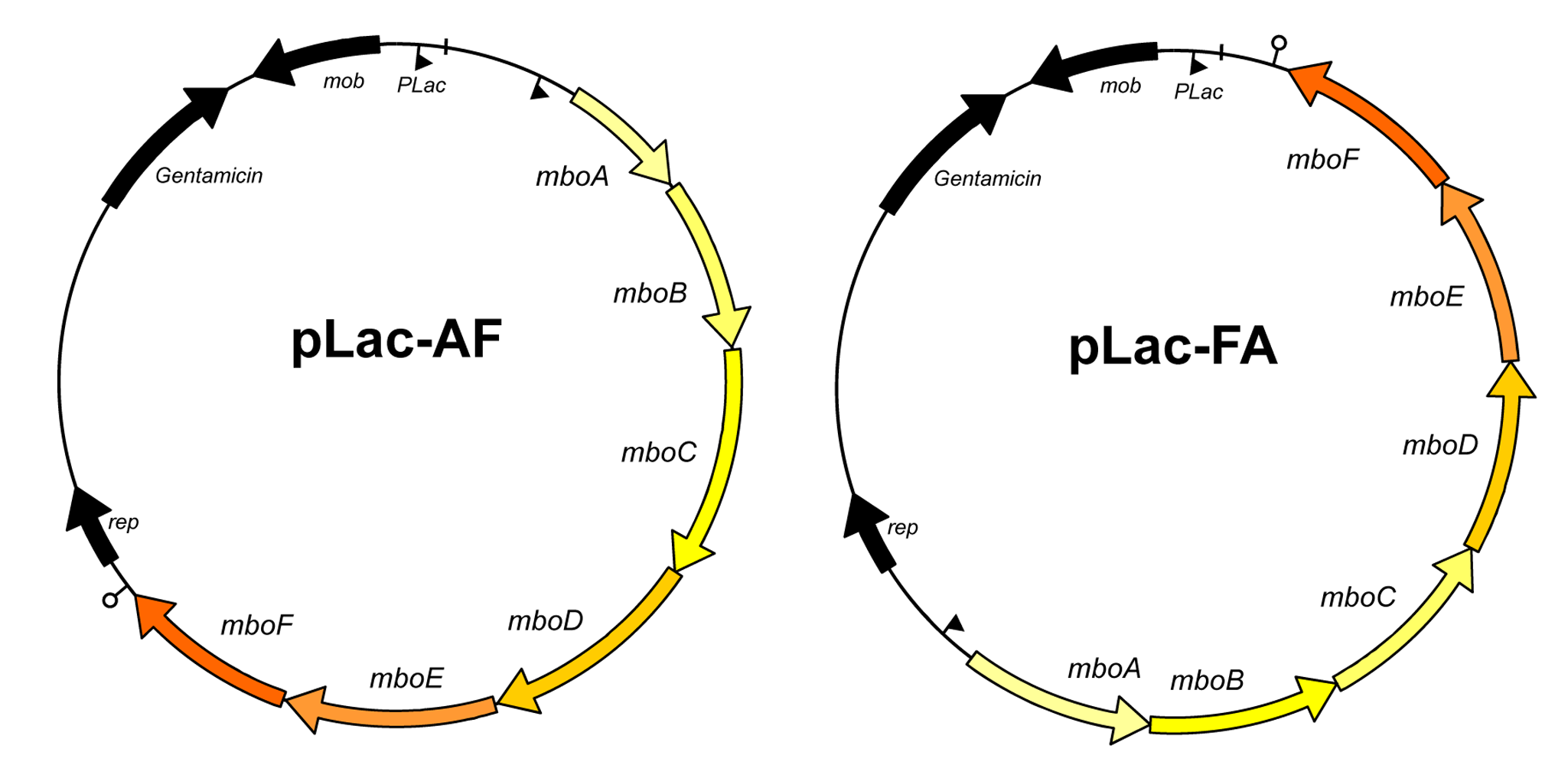

Supplement: Figure S3 — Construction of the pLac-AF and pLac-FA vector derivatives from pBBR1MCS-5. The complete mbo operon, including the regulatory sequences (putative promoter and terminator), was cloned into both vectors. In pLac-AF, the mbo operon is under the control of the PLAC promoter with constitutive expression in Pseudomonas spp. and the own promoter of the mbo operon, whereas the pLac-FA vector is affected by only the own endogenous mbo operon promoter. (TIF) [file pone.0036709.s003.tif]

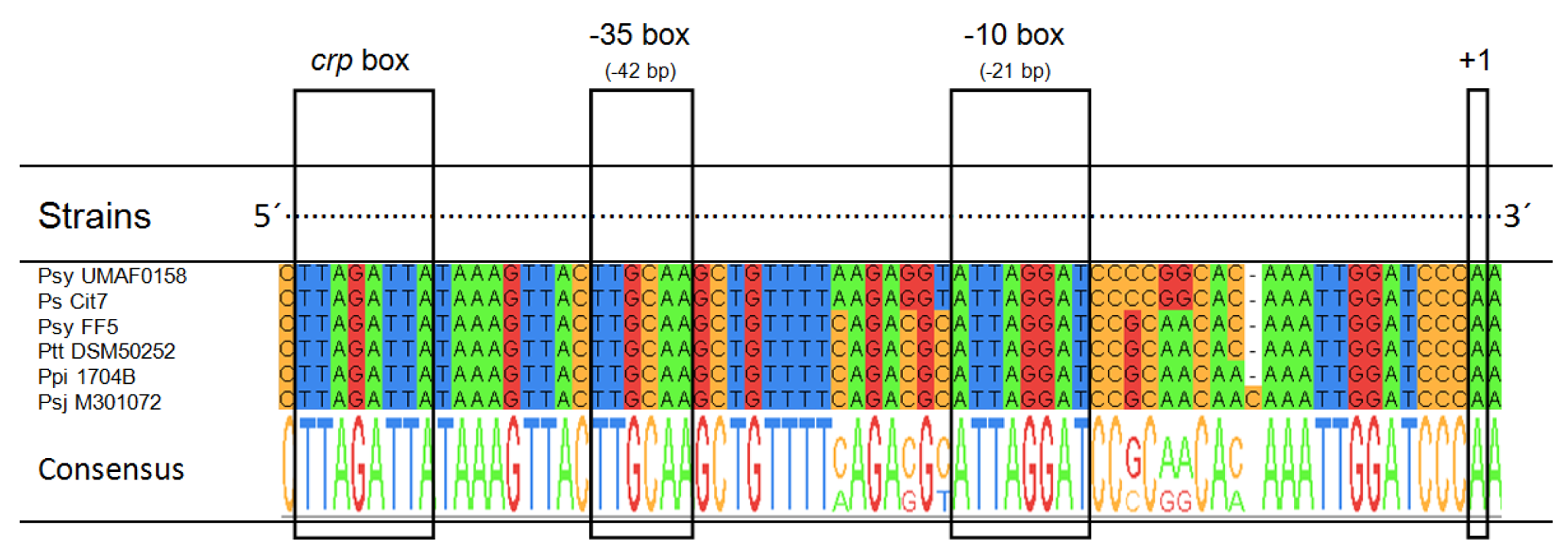

Supplement: Figure S4 — Comparison of the P mboI sequence motif between different P. syringae strains. This alignment was analysed using Jalview software. A summary of the tendency of each nucleotide to hold each position is represented under the alignment as a consensus sequence. The predicted -10 (position 21), -35 (position 42) boxes and crp box are marked in solid line. (TIF) [file pone.0036709.s004.tif]
