# Supplementary material for: The mbo Operon Is Specific and Essential for Biosynthesis of Mangotoxin in Pseudomonas syringae
Source: PLoS One. 2012 May 17;7(5):e36709. doi: 10.1371/journal.pone.0036709 (PMC3355146; doi:10.1371/journal.pone.0036709)
Supplement: Table S1 — Primers used in mbo genes mutation experiments, amplicons containing an internal fragment of each gene, were cloned in pCR2.1 for mutagenesis by integration. (DOC) [file pone.0036709.s005.doc]

| **Primers** | **Sequence (5’--3’)** | **Amplicon (bp)** | **Gene amplified** |
| --- | --- | --- | --- |
| MutA-for | ATCCGCATTCACATGAACAA | 208 | *mboA* |
| MutA-rev | CCATGAGTTTCCAGGTTTCC |
| MutB-for | GTTGCGATCAGCACTTTTCA | 201 | *mboB* |
| MutB-rev | GCAGGTCAGGCTGTCGTAAT |
| MutC-for | CCCGACGACATCGGCTTTAC | 393 | *mboC* |
| MutC-rev | CAGCGTCTCTTTGGCCAGGT |
| MutD-for | GCCATTCACCTGGAAATCAT | 202 | *mboD* |
| MutD-rev | CATCATCAGAAAACCGGTGA |
| MutE-for | ACCTGTTCAAGGGGGAAGTT | 200 | *mboE* |
| MutE-rev | CGTCAGGTCCTTGTCAGTCA |
| MutF-for | ATAGAAAAGCGCCGAGTTGA | 208 | *mboF* |
| MutF-rev | GTCGGTCCTGATGAACCTGT |
