# Supplementary material for: The mbo Operon Is Specific and Essential for Biosynthesis of Mangotoxin in Pseudomonas syringae
Source: PLoS One. 2012 May 17;7(5):e36709. doi: 10.1371/journal.pone.0036709 (PMC3355146; doi:10.1371/journal.pone.0036709)
Supplement: Table S2 — Primers used in RT-PCR experiments. NC1 and NC2 correspond to non-coding adjacent region upstream to mboA gene. (DOC) [file pone.0036709.s006.doc]

| **Primers pairs** | **Primers** | **Sequence (5’--3’)** | **Amplification region** | **Amplicon (bp)** |
| --- | --- | --- | --- | --- |
| 1 | NC1-for | GATGTGCTCAAGGCAATC | NC1 | 507 |
|  | NC1-rev | ACACAGTAAGTGCCACGC |  |  |
| 2 | NC2-for | GCGTGGCACTTACTGTGT | NC2 | 530 |
|  | NC2-rev | CCAGGATATGACGCTTGT |  |  |
| 3 | A-for | ACAAGCGTCATATCCTGG | *mboA* | 323 |
|  | A-rev | GCTGTACTTGTCGCCGTA |  |  |
| 4 | AB-for | TACGGCGACAAGTACAGC | *mboA-B* | 399 |
|  | AB-rev | CTTGCACACTCTGCACAC |  |  |
| 5 | B-for | ACACTTGATGCACGACAC | *mboB* | 695 |
|  | B-rev | GTGTCGTGCATCAAGTGT |  |  |
| 6 | BC-for | ACACTTGATGCACGACAC | *mboB-C* | 264 |
|  | BC-rev | GATGACCTGACGCACCTT |  |  |
| 7 | C-for | GTCAAGCCGATCGATATG | *mboC* | 502 |
|  | C-rev | CCAGGTACTCACACAGCG |  |  |
| 8 | CD-for | CGCTGTGTGAGTACCTGG | *mboC-D* | 682 |
|  | CD-rev | CGCTACATTCCGGTACTG |  |  |
| 9 | D-for | CAGTACCGGAATGTAGCG | *mboD* | 658 |
|  | D-rev | GAGACTGCACGCTGCTAT |  |  |
| 10 | DE-for | ATAGCAGCGTGCAGTCTC | *mboD-E* | 501 |
|  | DE-rev | CAGGCTGGTTGATGATGA |  |  |
| 11 | E-for | TCATCATCAACCAGCCTG | *mboE* | 666 |
|  | E-rev | CGTCAGGTCCTTGTCAGT |  |  |
| 12 | EF-for | TCTGGATCGATCAGAACG | *mboE-F* | 437 |
|  | EF-rev | CCAGTGATTGGACAGCAG |  |  |
| 13 | F-for | CTGCTGTCCAATCACTGG | *mboF* | 492 |
|  | F-rev | CAGTACCACATCGACGTG |  |  |
| 14 | FT-for | CACGTCGATGTGGTACTG | *mboF-T* | 515 |
|  | FT-rev | GAATATGCCAGCGGATGT |  |  |
